# Art for Ages: The Effects of Group Music Making on the Wellbeing of Nursing Home Residents

**DOI:** 10.3389/fpsyg.2020.575161

**Published:** 2020-11-26

**Authors:** Paolo Paolantonio, Stefano Cavalli, Michele Biasutti, Carla Pedrazzani, Aaron Williamon

**Affiliations:** ^1^Department of Research and Development, Conservatory of Southern Switzerland, Lugano, Switzerland; ^2^Centre for Performance Science, Royal College of Music, London, United Kingdom; ^3^Department of Business Economics, Health and Social Care, University of Applied Sciences and Arts of Southern Switzerland, Manno, Switzerland; ^4^FISPPA Department, University of Padua, Padua, Italy; ^5^Faculty of Medicine, Imperial College London, London, United Kingdom

**Keywords:** fourth age, health, music, nursing homes, older adults, wellbeing

## Abstract

In many countries, life expectancy has increased considerably in past years, and the importance of finding ways to ensure good levels of wellbeing through aging has become more important than ever. Arts based interventions are promising in this respect, and the literature suggests that musical activities can reduce isolation and anxiety and foster feelings of achievement and self-confidence. The present study examined the effects of group music making programs on the health and wellbeing of nursing home residents in Southern Switzerland. A team of professional and student musicians delivered 10 weekly music sessions in four nursing homes, focusing on singing, rhythm-based activities with percussion instruments, and listening to short, live performances. 22 participants (16 women and 6 men, aged 72-95 years, mean 83.6, SD ± 6.9) were recruited to take part in the study and were interviewed after the last music session. The data were analyzed with thematic analysis to investigate how residents experienced group music making and its effects. The findings show that the music programs were beneficial for residents’ wellbeing. Music plays an important role in their lives, both in their pasts and presently, and being involved in musical activities offers engagement and novelty in daily life, providing learning opportunities and facilitating interpersonal relationships. Moreover, these results were due to interactions with the musicians involved. Residents particularly appreciated the opportunity to listen to live performances as part of the sessions. This study suggests that nursing home residents value music and that music based interventions play an important and direct role in enhancing their wellbeing.

## Introduction

The global population is becoming older, and life expectancy is increasing in many countries (World Health Organization, 2015). Consequently, there is a need to develop and provide effective interventions to improve the wellbeing of those in later life. In this context, it is necessary to consider the needs of those in the fourth age, a final stage of life which is generally characterized by frailty, dysfunctionality and multimorbidity (Baltes and Smith, 2003). According to the World Health Organization, arts-based interventions play a crucial role in this regard, as they can concurrently impact physical and mental dimensions at both social and individual levels (Fancourt and Finn, 2019). Engagement with the arts can be active or receptive. In the first case, the focus is on *artistic processes*, such as creation, co-creation and performance, while in the second, the focus is on *artistic products* with the participants supported in approaching, understanding and appreciating artworks (Fancourt et al., 2014; Tymoszuk et al., 2018). For music, active engagement can include activities such as singing, playing instruments, and song writing, while receptive engagement implies activities aiming to facilitate access to and the appreciation of musical works or listening to live or recorded music. If we consider the studies focusing on the effects of musical activities in care environments for those in the fourth age, such as nursing homes, we find a rich literature arising from the field of music therapy (Finn and Fancourt, 2018). However, research on activities run by musicians that are aimed at facilitating access to artistic products and processes, without explicit therapeutic objectives, is still under researched.

There is overwhelming evidence that music is important for the wellbeing of older adults and is meaningful in later stages of life (Cohen et al., 2002). Research has shown that both listening to and making music can, irrespective of individuals’ musical abilities and backgrounds, reduce loneliness, promote positive affect and provide feelings of meaningfulness and purpose in life, contributing to delayed aging (Hays and Minichiello, 2005). Music is also beneficial for people affected by dementia and moderate cognitive impairment, improving their cognitive functioning, memory, mood, and general wellbeing (Biasutti and Mangiacotti, 2018, 2019; Ing-Randolph et al., 2015; Zhang et al., 2017; Van der Steen et al., 2018); positive effects have also been observed for their relatives (Särkämö et al., 2014).

With regard to music *listening*, older adults have been shown to use a variety of strategies to experience positive emotions, arising directly from music’s capacity to improve emotional functioning and identity (Laukka, 2006) as well as to provide pleasure and relaxation (Blood and Zatorre, 2001; Gabrielsson, 2001; Västfjäll, 2002; North et al., 2004; Menon and Levitin, 2005). Hays and Minichiello (2005) conducted a qualitative study involving 52 people, aged 60–92 years, which revealed that listening to music can entertain, provide aesthetically rewarding experiences, and promote positive feelings such as relaxation, cheerfulness, tranquility and excitement. Attending concerts is also beneficial for older adults. The participants in a recent study reported awe and gratitude for the music and performances on offer, and it emerged that this form of access to music can provide a route to rewarding emotions such as happiness and inspiration, to promote meaningful interactions and to reduce isolation (Costa and Ockelford, 2019). Numerous studies also suggest that music listening can reduce cortisol levels (Fancourt et al., 2014) and required dosages of drugs and sedatives (Conrad et al., 2007; Degli Stefani and Biasutti, 2016).

At the same time, music *making* has been shown to be beneficial to health and wellbeing in several ways. Research on singing suggests that it reduces anxiety and depression (Zanini and Leao, 2006; Sandgren, 2009), increases self-confidence and happiness (Lally, 2009) as well as emotional wellbeing and social functioning (Hillmann, 2002), and promotes positive self-assessment of overall health (Cohen et al., 2006). Furthermore, participants in singing activities show strong commitment and mutual support (Southcott, 2009; Southcott and Nethsinghe, 2019), and a study conducted by Cohen et al. (2006, 2007) revealed that people included in a singing group reported fewer visits to physicians, taking less medication, and experienced fewer health problems and falls compared with a control group. Singing can also enhance morale and reduce the risk of depression (Daykin et al., 2018), as well as promote cognitive abilities, improving attention, memory, concentration and learning (Clift et al., 2008). Similar results are reported in literature on playing instruments (Creech et al., 2013; Seinfeld et al., 2013; Perkins and Williamon, 2014). Studies based on keyboard instrument lessons have revealed that, compared with control groups, learners showed decreased depression and loneliness (Koga and Timms, 2001) and increased cognitive functioning (Bugos et al., 2007). These activities have been shown too to have benefits for cortical connectivity (Altenmüller et al., 2009), while Schneider et al. (2007) suggest that the therapeutic use of playing the piano or drums can improve dimensions of physical movement.

In recent years, several studies have considered the complexity of this field more broadly and have engaged mixed-methods approaches to understand better the perceived effects of music making programs for older adults. *Music for Life*, for instance, began in London in 2009 and explored the effects of singing in choirs, taking instrumental lessons and enrolling in music appreciation classes for 398 people aged 50–93 years. Using questionnaires, interviews, and focus groups to observe and understand how making music can improve older adults’ lives, the results showed positive effects on cognitive, social, and emotional dimensions as well as on mental and physical health, highlighting the need for such activities as well as the valuable training opportunities they offer for music providers (Varvarigou et al., 2012). Similarly, *Rhythm for Life* (Perkins and Williamon, 2014) involved approximately 100 people aged 50–74 years receiving one-to-one instrumental lessons, small-group instrumental lessons or creative music workshops. A mixed-method design employed standardized health questionnaires, in-depth interviews, and focus groups in order to observe the role of music on healthy aging and on hedonic and eudaimonic dimensions of wellbeing (Ryan and Deci, 2001). The results showed improvements in social, physical, and mental health. Furthermore, it emerged that the opportunity to learn music in particular was highly valued by older adults and that hedonic and eudaimonic dimensions were affected by a number of diverse factors, including the long-term desire to play an instrument, increased self-esteem due to their musical progress, and more social relationships not only with peer learners but also with the musicians providing the interventions (Perkins and Williamon, 2014).

Notwithstanding the amount and diversity of literature devoted to the effects of music in the lives of older adults, in most cases the average age considered has been around 70 years old; at the same time, if we consider the context of nursing homes, the literature focuses mostly on the effects of therapeutic music interventions (Hui-Chi et al., 2015; Degli Stefani and Biasutti, 2016; Brown Wilson et al., 2019). Consequently, the effects of music based interventions on people in the fourth age living in nursing homes are still under researched, and the present study aims to address this gap by exploring the impact of weekly music sessions in nursing homes delivered by higher education music students. In detail, we designed a 10-week program focusing on group music making, where students acted as facilitators and, in order to welcome residents and introduce new works, as performers. The musical activities focused on promoting the engagement with music *per se*, and participants were free to choose at any time whether to sing, play, or simply listen to others. This approach is informed by the notion of *active aging* and embraces the ideas promoted by the World Health Organization to move from a “needs-based” approach to a “rights-based” approach in the context of long term care (World Health Organization, 2015). This attempts to ensure the highest quality of life possible for nursing home residents, who are seen as citizens whose rights should be preserved and whose social participation, and thus also access to art, should be encouraged.

Using semi-structured interviews, we investigated, firstly, how residents in nursing homes experience the program of group music making activities and, secondly, what perceived effects these activities have on their wellbeing. Moving from the assumption that health implies more than the absence of illness and includes the presence of positive wellbeing indicators (Sigerist, 1941; World Health Organization, 1948; Ryff and Singer, 2003), our research questions were addressed through the lens of the complete state model of health, which considers health and illness as “correlated unipolar dimensions that, together, form a complete state of (mental) health” (Keyes, 2005).

The notion of wellbeing considered in this study is based on the PERMA model (Seligman, 2011), a theoretical framemork including the following five dimensions: Positive emotions (such as joy, serenity, relief and the like), Engagement (referring to the concepts of flow and loss of self-consciousness), Relationships (referring to satisfying social interactions), Meaningfulness (connected to individuals’ identity and to the sense of purpose in life), and Accomplishment (related to mastery, competence, and learning). In recent years several studies have explored the effects of music on wellbeing using this lens (Cromm, 2015), focusing on professional musicians (Ascenso et al., 2017), community choir participants (Lamont et al., 2017), and students in school contexts (Lee et al., 2017). Considering the paucity of contributions focusing on the potential of music in nursing homes, an interdisciplinary project, *Art for Ages*, was designed to examine the effects of group music making on the health and wellbeing of residents in nursing homes. Using a qualitative approach, the aim of the present study was to investigate whether this activity was meaningful and engaging for residents and to observe whether it promoted desirable emotions, social interactions and a sense of achievement.

## Materials and Methods

### Study Design

This study arises from *Art for Ages*, an interdisciplinary project run between April 2016 and January 2017 examining the effects of group music making on the health and wellbeing of residents in nursing homes in Southern Switzerland. Four teams of musicians delivered a 10-week program of group music making in four nursing homes, one per home, combining in each session singing, rhythm-based activities, and listening to short, live performances. An experienced workshop leader and a researcher (the first author) were part of all four teams to ensure consistency of delivery and interaction with participants. The total number of music students involved was 9, with 4–5 per team and some, on voluntary basis, involved in more than one nursing home. Students, who already possessed skills of advanced instrumental performance, were specifically trained to act as facilitators of the music workshops. The training consisted of being able to direct small groups of residents in singing, using the percussion instruments employed (boomwhackers, triangles, rattles, maracas, as well as common household objects such as spoons and graters), and to support learning in an appropriate and effective manner throughout the sessions. As a consequence of a quarantine for a serious influenza outbreak, the ten-week program was interrupted for seven weeks in one nursing home.

### Participants

Each music team delivered a public event in their assigned nursing home to introduce the musical activities proposed to residents. Each resident was offered the opportunity to participate in the program, and at the end of each session, the number of participants was noted by the staff of the home. The inclusion criteria for this study considered only residents who attended at least 8 sessions and who were able (in terms of their health) to be interviewed. Forty-one residents (34 women and 7 men, aged 72–100 years, mean 86.5, SD ± 7.5) met the first requirement, and from them, 22 (16 women and 6 men, aged 72–95 years, mean 83.6, SD ± 6.9) met both criteria.

### Procedure

Each group music making session lasted 45 min. The musicians performed some live music both at the beginning and during the sessions, which was designed to engage participants and to make their experience as enjoyable as possible. The program for each nursing home was refined by the workshop leader as the 10-week period progressed, and the repertoire included songs requested by residents. Even though each music team delivered its own program, the overall approach and musical activities were common to all programs and included agreed songs at the beginning and at the end, musical warm-up exercises, and the practice of a varying between new songs and the revision of previous songs. During the sessions, residents were able at any time to choose whether or not to take part in each song or exercise proposed by singing, by accompanying with the instruments reported in the study design, or by listening.

Data were collected through individual semi-structured interviews conducted by the researcher (first author), who was known to participants as a member of the music team. The interviews took place in Italian either in residents’ private rooms or in common spaces of the nursing home during the week following the last music session. Inspired by a phenomenological approach, the questions invited interviewees to evaluate the program, to talk about its effects on themselves and on their relationships with other people involved in the sessions, and to provide a self-evaluation of their musical abilities. The full list of questions is provided in Appendix 1. As a consequence of being involved as a musician throughout the program, the researcher was sensitive to the possibility of influence over participants’ answers and encouraged each person to express their ideas and opinions freely, inviting them to describe in depth specific situations and offering space for unexpected contents and digressions (Kvale and Brinkmann, 2009). The interviews were audio recorded with permission of the participants and fully transcribed by the researcher.

The study was granted ethical approval by the Ethical Committee of Canton Ticino (CE 3030-2016-00193).

### Data Analysis

The first author transcribed verbatim the interviews, and then thematic analysis (Braun and Clarke, 2006) was applied in collaboration with a researcher outside the project. To reconstruct to the fullest extent possible the chronemics and paralinguistic contents (Gorden, 1980) of each interview, and recognizing the potential limits of transcripts in terms of reliability (Poland, 1995; Kvale and Brinkmann, 2009), the audio recordings were constantly referred to throughout the data analysis. All the transcripts were considered and manually coded with the view of answering the research questions. The codes were organized into categories, themes and sub-themes. This material was submitted to the other members of the research team and all elements considered ambiguous or redundant were discussed and modified accordingly. Once agreement was reached, each interview was re-analyzed and annotated by the first author and discussed with the researcher external to the project according to the following criteria, determined by the coding scheme:

• role attributed to music,
• changes of the role of music due to the program,
• nature, intensity and duration of the effects of the sessions,
• overall evaluation of the program,
• interpersonal relationships,
• experience of making vs. experience of listening to music during the sessions,
• peculiarities (e.g., relevant exceptions, individual differences, critical issues).

Grouping rich accounts of data in this way allowed for a deeper and more detailed understanding of the complexity and the nuances implied by each criterion. In order to reconstruct individual experiences of the program, each interview was then analyzed again observing for each participant the interactions of these criteria. This analysis was discussed within the research team and led to the elaboration of a summary of the results.

## Results

As shown in Table 1, six themes and twenty-three sub-themes emerged from the analysis. To protect the anonymity of the participants, false names are reported.

**TABLE 1:** Overarching groups, themes, and subthemes arising from the analysis of residents’ interviews following the 10-week group music making program.

Overarching groups	Themes	Subthemes
**Effects of the**	Positive effects	Happiness.
**program**		Fulfilment of the need for novelty and engagement. Activation of autobiographical connections. Anticipation of the session.
	Negative effects	Melancholy provoked by music. Disappointment based on expectations.
**Experience of the program**	Role of music in residents’ lives	Interest in music and its positive effect.
		Ability to listen to music autonomously.
		Relationship with people who were active in music.
		Relationship with singing.
		Relationship with instruments.
	Learning and discovery	Pleasure in producing sound.
		Enlargement of musical knowledge. Enhancement of engagement in music listening.
	Interpersonal	*With other residents*
	relationships related	Opportunities to meet.
	to participation in the	Mutual support.
	program	*With the music team.* Gratitude. Familiarity.
		Intergenerational encounters.
		Encounters with new people.
	Recurring concerns in	Feelings of isolation.
	residents’ daily lives	Doubts about cognitive abilities.
		Lack of novelty and engagement.

### Effects of the Program

The effects described by participants belong to the dimension of mood and emotions, and taking part in the program evoked happiness and other positive feelings. It emerged from interviews that this was mostly due to how the overall program was carried out, rather than to its specific aspects.

*[The program] had very positive effects on me because I liked very much everything you did. [*…*] I was very happy! I was really happy! [Every time] I spent a lovely lovely lovely hour. (Sarah, 90)*

These positive feelings may have been linked to the novelty introduced by the program in the everyday life of residents or to the activation of ties with their own life experiences.

You came here and made us spend an hour in happiness, with something different from what we do every day. It is a wonderful thing to have something engaging us and able to change our day. (Rosie, 83)

I have been happy because [the program] is something enabling me to be myself again, namely to listen again to music that I did not listen to for a long time. (Tina, 86)

*Every time I saw you [the researcher] coming with your double bass*…*I liked it! I was seeing my father [who was double bass player]! (Peter, 95)*

For these reasons, the session day was anticipated with happiness.

*I was always waiting for [*…*] Wednesdays, then I was waiting for 10.30. It was almost a sort of romantic relationship. (Miles, 92)*

We were looking forward only to Fridays because it was the lesson day! It was something enjoyable for us, and when you like something, you are looking forward to that day. (Lucy, 75)

In only two cases, the effects on mood were not positive. In detail, it emerged that memories recalled by music provoked melancholy for a participant, while in two cases expectations were not met because the goals of the program were unclear or because its contents did not develop the participant’s own musical competences.

My character is a little melancholic. If I listen to music, or maybe if you get out unintentionally some music related to my youth, this makes me sad. (Maria, 77)

*I found [the program] almost pointless*…*because it left me indifferent. I just laughed while we were using the basket making “tac tac, tic tic.” And I was wondering “What is the point?” (Robert, 85)*

### Experience of the Program

The analysis revealed that the aforementioned effects are related to four themes. The first is connected to the importance of music in the life of residents, the second with the learning opportunities offered by the sessions, the third with the interpersonal relations experienced during the program, and the fourth to the usefulness of the program in coping with recurrent feelings and concerns.

#### Role of Music in Residents’ Lives

Although the interviews revealed diversity among residents regarding their musical backgrounds and their involvement in music, it emerged almost unanimously that music was an important and valued art form, able to induce emotions and affect mood.

I like everything related to music and to musical instruments. It makes me happy! I think it benefits the spirit. For me, music is life! (Ingrid, 96)

I think music is very important for elderly people. If you listen to a song or sing, you feel younger, you feel more alive. (Dawn, 83)

*For me music is art, so I feel grateful when it is available. Everything related to art and able to reach your hearth is always welcome*… *If we take art away from life, what’s left? I could say music is a relevant part of my life, definitely, even though I never played an instrument. (Tina, 86)*

Music tells me something! It really does that! I am not indifferent to music, but I am not used to sitting down just to listen to music. (Robert, 85)

This interest in music manifested itself through the regular use of radio and similar devices. However, listening to music autonomously was not always completely satisfying because the impact on the mood may have been negative.

Every night I always listening to Rete2, always hoping to listen to good music to take me to sleep. Music gave a lot to me! And it still does! I can’t read anymore, and often I don’t like television broadcasting. (Ingrid, 95)

There are songs that give me joy and songs that give me sadness. (Rosie, 83)

Besides the benefits associated with music listening, music was also relevant because someone important in their life had been actively involved in music, or because they themselves used to sing.

*My brother worked in an orchestra*… *In Switzerland we have the Suisse Romande Orchestra, which broadcasted concerts on the radio, and he worked there. Consequently, some interest in music remains. (Maria, 77)*

I still remember singing with my husband when traveling by car. When leaving for holidays, we would sing mountain songs along with our children. (Lucy, 75)

Beyond that, singing was still an important resource, and residents who used to sing maintained their music making because they were, to some extent, aware that it was beneficial and kept negative feelings away. It emerged that some residents accessed these benefits irrespective of their abilities, while others, concerned about their limits in singing, would sing only in specific conditions.

For me, music is a great support for the spirit! While you are singing you don’t have sad thoughts, or perhaps you do, because you remember someone you loved. Singing is good for me, I sing gladly. (Ingrid, 95)

*When you sing a song, even though you didn’t compose it, you sing it gladly because it is something that awakens you. It wakes the body and soul. Furthermore, it keeps away some thoughts like: “Now I just have to wait to die”*…*no! (Dawn, 83)*

If there is a singing group, I also sing because I still have the voice to sing. But I don’t sing alone! (Fiona, 78)

I always sang at home. But now I don’t anymore have my past voice, and this make me laugh because I think: “Gosh, if someone heard me singing he would throttle me!” But I sing anyway! (Amy, 79)

Cases reflecting active involvement with musical instruments referred only to the distant past. Nevertheless, it emerged from the interviews that this kind of musical activity, no matter whether actual or contemplated, was still in the thoughts and desires of residents.

*I began to play piano in early childhood. My sister played violin, and we performed duets*… *Now I can’t play because my hands don’t work anymore. Furthermore, here it is impossible to play piano. (Ingrid, 95)*

*I always liked music. Sadly, I wasn’t able to study it. I liked the piano, but I could not afford to study it*… *In my village a guy owned an accordion, and sometimes he allowed me to play, and I quickly learned to play the melody by ear. Afterwards, you know, you leave [the village] for work, and you don’t stay in touch anymore. (Dawn, 83)*

After all, I miss an instrument. My mother was right: “You could at least study piano!” Now it is too late (smiling). (Miles, 92)

#### Learning and Discovery

The positive emotions reported by participants are also related to the numerous learning opportunities offered by the program.

Taking part allowed residents both to receive information and instructions from the musicians and to manipulate the instruments used. With reference to the latter point, the opportunity to play percussion instruments, exploring the diverse sounds in a friendly and playful environment, provoked curiosity and fun.

*Well, I liked the triangle very much, I really liked having that small object to beat! I also liked the small drums and the bells*… *We wanted to try them, and we really liked it! [*…*] Playing those small instruments was really nice and enjoyable. (Sarah, 90)*

*We had fun, like a child provided with sticks and allowed to play*… *(Robert, 85)*

The musical interaction within the group was central to providing pleasure in producing sounds. That was engaging because, on the one hand, this interaction required attention and, on the other hand, the mistakes and the musical imperfections were funny and intriguing.

*I had also laughs, when someone made this and another made that [she mimes two uneven gestures]*… *It happens when one gets old, but it was funny for me. (Paul, 72)*

Having to concentrate was lovely too, whether we were playing drums or whatever else. It was also funny. (Jane, 81)

Musical activities were experienced with happiness and engagement especially because they were considered ‘proper’ learning opportunities, relating to very diverse aspects connected to music. The interviews reveal positive thoughts about the overall design of the program and the progress made session after session. A resident expressed disappointment as the program ended without any special activity.

*For us it was a sort of learning through playing. I mean: as you teach children how to play, you also teach the elderly how to play*… *At the beginning we took it [the program] as a joke, as a way to have laughs. But, little by little, we understood that while having fun, we were learning something. (Lucy, 75)*

*At the beginning we did our tasks poorly, while now, at the end, we do them much better!*… *I just observed that*… *I was used to chatting a little with all of you [members of the music team], and I felt gradually more secure. Yes*… *making music together reduced the awe I felt, definitely*…*and it made everybody happier! (Carol, 75)*

*I expected who knows what!*… *It was the same from the beginning to the end*… *We wanted something for the conclusion!*… *But it was good to take part in this program. (Robert, 85)*

The combination of musical instruments and playing music with common objects was especially appreciated and represented a surprising revelation and discovery.

I didn’t believe one could make music using those [objects and small instruments], but it is possible. I heard, and I am happy for having observed and learned that. (Emily, 88)

*You taught us that actually there are situations where one can make music even with simple objects*… *Let’s consider the grater, for instance: who knew that with such a tool you could make music? Nobody!*… *And then we learned also that. (Lucy, 75)*

If we consider more closely only the musical aspects, participants referred to their improved ability to keep rhythm, their understanding of processes related to music creation, and their new capacity to approach and appreciate unknown repertoires. The time devoted to anecdotes and information related to the biography of famous musicians was, in turn, appreciated.

Now I would evaluate 8 [grade 1 to 10, related to the ability to keep rhythm] because I just learned what it means to keep rhythm. (Carol, 75)

*It was really very interesting!*… *[A way to] discover that music is something different to what I believed. I could not understand how you musicians do it*…*and then, I learned how one can make music! (Amy, 79)*

Russian music, in particular, was unfamiliar for me, and I liked it very much. (Peter, 95)

Every time we discussed something about the life of Mozart or somebody else, that was nice too! (Elisabeth, 80)

In the rhythm-based activities the learning opportunities were generally welcomed regardless of familiarity with the songs and activities proposed. However, the inclusion of unfamiliar songs in the singing-based activities required some effort for participants.

*Dealing with the songs you know is simpler. You go in effortlessly*… *It is a little more tough to deal with learning new songs because you have to study, you have to read*… *(Dawn, 83)*

*I preferred the songs I already knew. To sing the other [unfamiliar] songs, you have to learn them.[*…*] When they [the musical team] teach you, when they engage you in singing German songs, you never learn them completely, and this is a problem. I know, they give you also the sheet (with lyrics), but it becomes a little difficult! You have trouble. (Frank, 91)*

Taking part in the program had effects also on listening to music, making this activity more engaging.

*Before, I was used to listening to music ‘en passant’. Now, I listen to it differently*… *with more attention. Now I try to listen a little more deeply, and I find again information I was taught at school. (Paul, 72)*

*I think that, now, I listen more to the music than to the lyrics. I mean, before, I was focused mostly on lyrics, now instead I am almost more focused on music than on lyrics*… *Before I listened to lyrics but not to the music! These days I think: “Gosh, this music is wonderful!” (Lucy, 75)*

#### Interpersonal Relationships

The effects reported by participants were related to interpersonal relationships in two respects: relationships with other residents and with members of the music team.

##### Relationships with other residents

In the context of nursing homes, the program constituted an opportunity to meet, consolidating relationships between residents and offering conversation starters, particularly about musical topics.

I talked with other residents! Even last Friday, with the lady close to me. We talked about music and operatic arias. (Amy, 79)

I talked mostly with my friend. Yes, we talked a lot, and we are very happy about the whole program. I would not say I previously had a direct relationship with other [residents]. But everybody knows better each other through making music! Definitely! (Carol, 75)

In some cases the program promoted the mutual support and the sharing of impromptu and spontaneous musical activities.

*A person I became friends with, who also participated in the program*, *liked it too. She has some problems with memory, and she asked me: “Remind me when the music session is so I can join you”, and last time she went before me and kept the place for me. She liked a lot the program and taking part in it together made her happy. (Sarah, 90)*

We all felt well, and sometime, in the evening, we sang that ritual opening song “Siamo qui siamo tutti qui” (singing). (Dawn, 83)

Nevertheless, the data also reveal that, due to individual attitudes or to the recent relocation to the nursing home, taking part did not affect their relationships with other residents.

The relationships [with other residents] remained the same. After all, I am not a chatterbox. When I come across somebody I salute them; I chat with them. (Miles, 92)

Well, it’s not been that long that I moved here, barely a year. Then I talked very little with other residents (with a sad tone). (Maria, 77)

The interactions occurred during the sessions, and the concentration demanded by the activities also brought to the surface small frictions between residents.

*There was a lady in the group, odious and even ugly, who was always out of time. And when I looked at her, I went out of time too*… *When one makes music and brings the time, you can’t go out against the time! (Miles, 92)*

I don’t like people who always want to prevail. Unfortunately [in our group], there was a lady with this flaw, and this gets me nervous. She always prevails, always! Now I let go. Why should I get nervous? (Rosie, 83)

##### Relationships with the music team

The encounters and interactions with music team were often positive. The interviews reveal feelings of warmth and gratitude.

*I bonded with each of you (smiling); you were lovely and kind. Did you notice I came to greet you at the end of each session?*… *Honestly I felt very well with you. (Sarah, 90)*

First of all I was delighted to be acquainted with you, people taking care of me and my peers. (Emily, 88)

The musicians avoided a formal and detached approach with residents, and this was unanimously appreciated. The friendly, serene atmosphere established throughout the program promoted empathy and triggered ties with residents’ life experiences. The encounter between different generations had an important role in this sense.

I liked [the relation with musicians]! That reminds me of the time when my son was in high school, and I felt as I could feel their problems and share with them my problems. Do you know what I mean? (Maria, 77)

The guys were very, very nice! They made themselves available to make us able to do a good work, and they achieved that! They were very lovely, yes – wonderful and lovely. The flower of youth! (Elisabeth, 80)

Due to personal habits or individual character, a few residents taking part in the program did not experience meaningful interactions with music team.

*I am not very sociable*… *I made my music, I followed the instructions, and that’s all. (Paul, 72)*

Besides personal interactions, the arrival at each nursing home of the music team offered an opportunity to see new faces and figures. Residents found it interesting to observe the personal and musical interaction within the team and appreciated the musicians’ commitment and energy in doing their work.

I liked it very much because it was clear that there was a strong chemistry between you musicians and that you enjoyed making music together. (Sarah, 90)

I liked that opening ritual song because I thought: “It also is a sort of welcome”. [It expressed] your pleasure, the pleasure perhaps you had in the same way we had. (Elisabeth, 80)

#### Recurring Concerns in Residents’ Daily Lives

The interviews offered room for thoughts related to the everyday life in the nursing homes and to the meaning of musical activities within this context. In this regard three subthemes emerged, offering further elements to understand the effects of group music making and how it was experienced. First of all, the opportunity to take part in the program represented a valuable opportunity to limit the feelings of isolation perceived when living in a nursing home.

*Your idea is very good, in that way we don’t feel completely lost, we [see] more people and [we have] more interactions*… *Here many [residents] cannot even move from their chairs. (Emily, 88)*

*In my opinion music is extremely important for the elderly. If you listen to a song, or if you sing it, you feel younger, you feel more alive*… *Do you know what I mean? Because in being just nothing, one regresses. (Dawn, 83)*

For residents strongly interested in specific music genres, the feeling of isolation was explicitly related to their musical tastes, as it may not have been possible to share with anyone their passion for specific repertoires.

*I like very much classical music too, but it’s not easy! Because*…*not everybody is used [to that]. I am talking about people living here*… *Here we are all old! Senile! (She laughs)*…*Believe me, it’s not easy to live here. (Ingrid, 95)*

*I think people living here are not very musically cultivated. The only music they enjoy are the ditties, “La bella lavanderina” [a folk song], and that’s all*… *I like any kind of music, but to listen to a recording is not the same. With you musicians one can talk and share impressions about music and about the feelings it provokes. (Tina, 85)*

As we have already seen, the program was to a significant extent experienced as a learning opportunity. Nevertheless, some residents revealed doubts about their own cognitive abilities.

I would like to study, but perhaps the brain regresses. (Dawn, 83)

[With regards to the possible changes and improvements of the program] I would not change it so much, no. We are quite limited, in the truest sense of the word. (Carol, 75)

Beside the thoughts related to learning, the interviews reflected thoughts related to novelty and engagement, and in particular attention and imagination were considered under stimulated by some.

[The program] was positive mainly because one learns to pay attention, which is not something we have to do so much here. (Carol, 75)

I think that [during the sessions] our imagination is very stimulated. The program supports our imagination. (Miles, 92)

## Discussion

**FIGURE 1:**
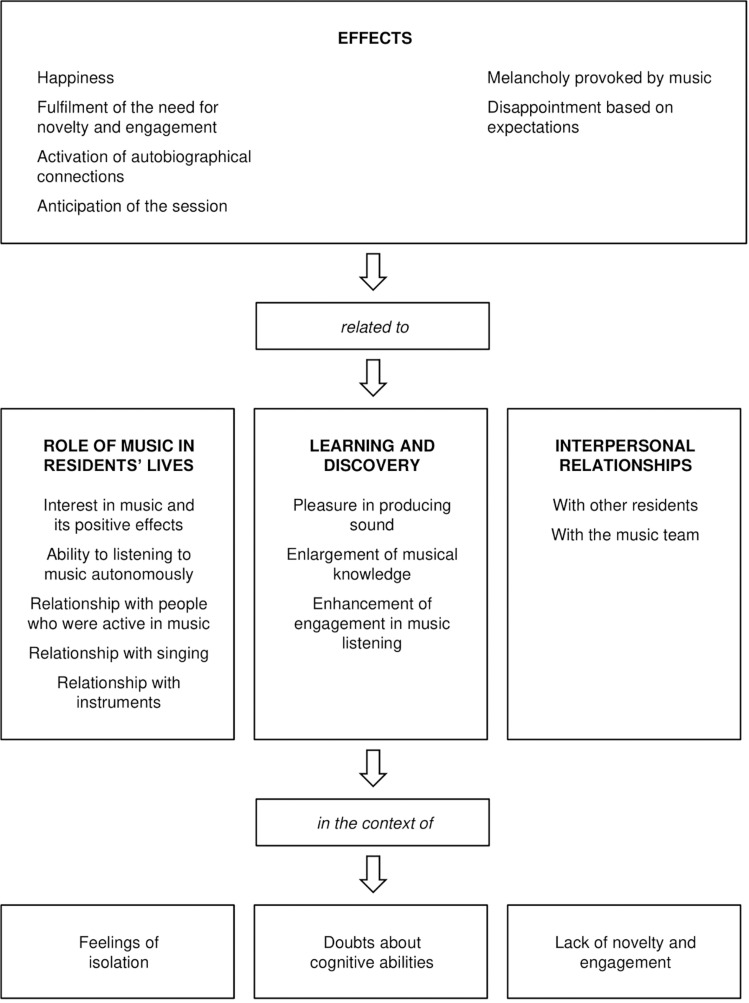
Interactions between themes and subthemes arising from the interviews.

This study suggests that the program of group music making had effects on the mood and emotions of participants and, to some extent, enhanced autobiographical memories. These effects were related to three elements: participants’ interest in music, their appreciation for the learning opportunities included in the program, and the interpersonal relationships promoted by it. The results are aligned with those of other studies (Hays and Minichiello, 2005; Creech et al., 2014; Perkins and Williamon, 2014; Yap et al., 2017) and are novel as they reflect the context of nursing homes, as participants reported also feelings of isolation, doubts about their own cognitive abilities, and a lack of novelty and engagement. The overall picture emerging confirms the idea that the benefits perceived from the engagement with music derive from complex processes, which include diverse dimensions largely influenced by idiosyncratic needs and circumstances (Perkins et al., 2020). In this case, as illustrated in Figure 1, the results suggest that this program has had mainly positive effects as it facilitated access to something considered important, offering learning opportunities and promoting interpersonal relationships in a context where novelty and engagement are sometimes missing.

Residents reported mostly positive effects, and this was due to the fact that the program offered, in an environment where they sometimes lack a sense of novelty, a combination of receptive and active engagement with something they considered important. Thanks to the ability of music to trigger memories and to strengthen links with one’s identity and biography, taking part in the program represented a valuable opportunity for residents to stimulate and to value their cognitive abilities. Furthermore, the collective dimension of the program facilitated interpersonal relationships. This element seems particularly important considering the feelings of isolation reported by interviewees and often experienced in the context of nursing homes (Costa et al., 2018a,b). In this regard, it emerged that the presence of music students, who were seen as young yet highly skilled people, both as artists and music facilitators, had a crucial role. This overall picture is in line with the recommendations of the World Health Organization in terms of the quality of life in long term care, which stresses the importance “to ensure that a person who is not fully capable of self-care can maintain the highest possible quality of life, according to his or her individual preferences, with the greatest possible degree of independence, autonomy, participation, personal fulfillment and human dignity” (World Health Organization, 2002).

A deeper analysis suggests that each of the three components of *group*, *music*, and *making* had an important role in the residents’ experiences, and the majority of themes and sub-themes were related to a combination of at least two of them.

The relevance of *music* was crucial: participants were delighted to take part in the musical activities. In some cases, they waited the whole week for the session, and by attending it they strengthened links to their memories and identities. Both listening to and making music improved mood and stimulated attention and imagination, in agreement with other studies (Hays and Minichiello, 2005; Laukka, 2006; Lally, 2009; Costa et al., 2018a,b) that also show that music is often considered beneficial to wellbeing (Perkins et al., 2020). In addition, the program elicited positive emotions and represented a pleasant novelty because it offered the opportunity to listen closely to high quality live musical performances and to approach music making supported by and interacting with skilled musicians. In this context, residents appreciated the opportunity to discover works and composers previously unknown, satisfying their curiosity and evoking experiences from their school days. Consequently, and in line with previous studies (Hays and Minichiello, 2005; Perkins and Williamon, 2014; Costa and Ockelford, 2019), music was seen as something engaging and understandable, and from the interviews it emerged that the opportunity to increase one’s cultural background and to acquire new knowledge was greatly appreciated. While the positive effects of familiar or preferred music on older adults are well documented (Costa et al., 2018a; Southcott and Nethsinghe, 2019), as well as the tendency to determine musical tastes in earlier stages of life (Zimprich and Wolf, 2016), our findings related to the curiosity and the enjoyment in discovering new repertoires invites a deeper consideration of the role and the possible benefits of music in the latest stages of life.

The dimension of *making* played an important role too, as residents were able to “make” for themselves, having fun in experimenting and being allowed to explore in a friendly, informal and non-judgmental environment. That seems to confirm the relevance of learning opportunities for older adults (Formosa, 2002, 2012) and the crucial role of facilitators (Hallam et al., 2015) in a context that may be perceived as lacking in stimuli and where individuals may doubt their own abilities. Considering more closely how singing and drumming were experienced, it emerged that learning new lyrics can be in some cases challenging and demotivating, especially with songs in foreign languages. At the same time, singing popular songs linked to participants’ youth was particularly engaging, and residents born in Spain or in the German-speaking regions of Switzerland appreciated the inclusion in the repertoire of some songs in their mother tongues. With regard to percussion instruments used, interviews revealed that the opportunity to use common household objects rather than proper instruments stimulated the curiosity and imagination of residents, both in terms of achievable sounds and of the atmosphere and moods evoked. Furthermore, coupling common objects with proper musical instruments contributed to creating a cheerful and informal environment, encouraging some participants to overcome their shyness.

The dimension of *group* was, in turn, essential, and this program made it possible to promote two distinct kinds of social relationships. On the one hand, the encounter with the music team and its individual members, who brought with them a strong element of novelty and excitement into the context of the nursing home, stimulated feelings of gratitude and familiarity. On the other hand, in many cases the dimension of *group* contributed to strengthening the relationships between residents themselves, as the program offered opportunities to meet, chat and offer mutual support in a fashion similar to that highlighted in the literature on community music and participatory arts (Lally, 2009; Varvarigou et al., 2013; Lamont et al., 2017).

Negative effects occurred only in a few cases. The capacity for *music* to modify the mood and elicit emotions instilled feelings of melancholy or enhanced depression. These effects, while confirming the importance of allowing individual choice in selecting music (MacDonald, 2013; Costa and Ockelford, 2019), stress the importance of including in such programs a wide range of musical styles, genres and repertoire. With regards to the combination of *making* plus *music*, a participant clearly expressed disappointment as the program did not include an overt climax or a special event at its end. This is a clear suggestion about how to improve such an intervention, and in recent years, several studies have indeed included performances (e.g. the *Rhythm for life* and *Music for life* programs).

**FIGURE 2:**
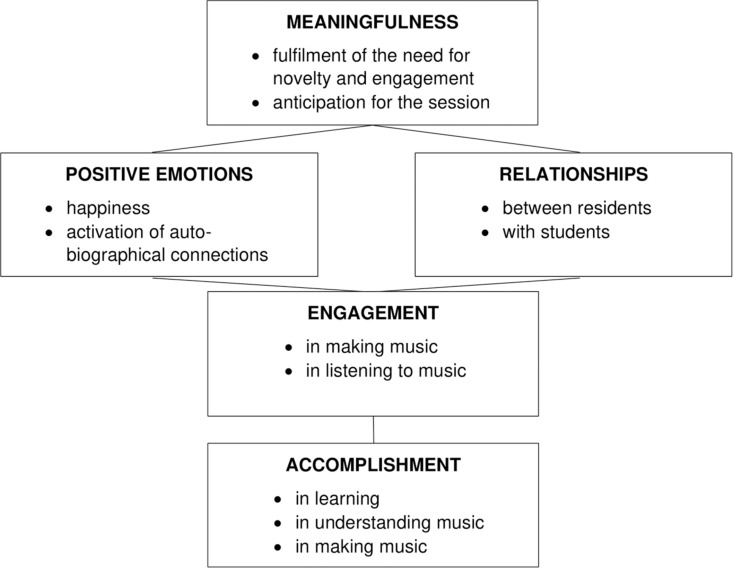
Relationships between the results and the five dimensions of PERMA model.

Considering this overall picture through the lens of the PERMA model (Seligman, 2011), our findings can be summarized as illustrated in Figure 2.

Our results resonate with the idea proposed in previous studies (Hays and Minichiello, 2005; Southcott, 2009; Lamont et al., 2017; Costa and Ockelford, 2019) that music has a *meaningful* role even in the late stages of life, as in its different forms of consumption it can trigger autobiographical memories, reinforce identity, and stimulate the most intimate and spiritual dimensions of individuals. In line with earlier contributions (Laukka, 2006; Varvarigou et al., 2012; Lee et al., 2017), the interviews also suggest that taking part in a music program provided *positive emotions* in the nursing home context, and that the collective dimension of the activities proposed increased in significant and valuable ways the *interpersonal relationships* of residents. Considering in detail how interviewees described their experience of the program, it emerged that the combination of musical content and interpersonal interactions made residents motivated and interested throughout the program, of which both the active and receptive components were considered *engaging.* Finally, due in particular to the active component of the program and to the supportive approach of the music team, it emerged that this kind of experience provided a sense of *achievement*, as residents reported improvements in their singing and rhythmic skills as well in their ability to understand and appreciate music.

The overall picture indicates that both making and listening to music can be engaging activities for nursing home residents, able to enhance wellbeing regardless the frailty and multimorbidity that can affect people in the fourth age. Furthermore, it emerged that music students can play a key role in these effects, as their participation provided both social interactions and aesthetic experiences considered meaningful by residents. These results are encouraging for the further work of this type, with three notable avenues for future initiatives. Firstly, it is important to invest in training programs for musicians that combine high skills both as music performers and as music facilitators. As shown here, this can help satisfy nursing home residents’ desires for music and to facilitate their access to and participation in artistic content and processes. Secondly, our exploratory study was confined to 10 sessions behind closed doors; carrying out longer programs, including some public events, may shed more light on the potential long-term effects of group music making as well as the impact on individual wellbeing in terms of the achievement and relationships dimensions of the PERMA model. Finally, further research is needed to explore the effects of music activities on the wellbeing of carers. Previous contributions focusing on hospital settings have highlighted benefits to healthcare professionals in terms of compassion, empathy, wellbeing, and reduction of stress (Crawford et al., 2015; Smilde et al., 2019), and such effects would be pertinent for those working in nursing home environments.

Some limitations of the present study should be acknowledged. Firstly, participation in the program was on a voluntary basis and this could suggest that the investigation included mostly those individuals who were particularly enthusiastic about social activities and about music in general. However, not all participants in the study felt that the musical activities, in their entirety, provided wholly meaningful experiences, as reflected in the remarks quoted in this article. Secondly, it is important to mention that the study did not compare group music making with other participatory activities; consequently, it remains unclear whether the effects reported could be achieved through other pursuits delivered in a similar fashion. Nonetheless, music making is physically, cognitively, emotionally and socially engaging and is typically present throughout the course of people’s lives. While this is not unique among interventions that could be employed in nursing home contexts, research suggests that music indeed plays a powerful role in life (Cohen et al., 2002; Hays and Minichiello, 2005), is a cost effective mode of engaging people (Fancourt et al., 2016), and is well suited to be applied across the full spectrum of health states, including those faced with impairments that may accompany the aging process (Matarasso, 1997; McLean et al., 2011). Thirdly, only individuals able to speak, and with unimpaired cognitive functioning, were recruited by nursing home staff for this study. Further research should be carried out that includes people with cognitive impairments associated with aging, such as dementia. In order to overcome the difficulties implied by cognitive impairments, ethnographic-informed approaches seem particularly promising (Smilde et al., 2014; Toye et al., 2014; Perkins et al., 2020), and future research may involve nursing home staff in the collection of both qualitative and quantitative data. Fourthly, the nursing homes involved in this research were in close geographical proximity (located within and overseen by the same Swiss cantonal institution) and regularly work in close partnership. It would be instructive for future research to reach across a wider range of homes, covering different geographical, socioeconomic and cultural spheres. Finally, a further point to consider is the risk of bias due to the fact that the first author was involved both as a member of the music team and as an interviewer. This point was carefully addressed both during data collection and analysis. Interviewees were invited to provide detailed descriptions of their experience while unexpected contents and digressions were welcomed and supported (Kvale and Brinkmann, 2009). This approach created an atmosphere of respect and trust which allowed residents openly to express critical remarks and perplexities related to specific aspects of the program, for instance about the schedule of activities or personal musical tastes. The results presented here have been evaluated, with caution, through a reflexive lens and the collaboration within the research team and with other researchers external to the project was extremely important throughout data collection and analysis.

In conclusion, our findings show that group music making was beneficial for the participating nursing home residents and impactful across the wider nursing home contexts. Music clearly played an important role in residents’ lives, both in the past and presently, and making music offered them engagement and novelty, providing learning opportunities and facilitating interpersonal relationships.

## Data Availability Statement

The raw data supporting the conclusions of this article will be made available by the authors in forms appropriate to ensure the anonymity of each participant.

## Ethics Statement

The studies involving human participants were reviewed and approved by Ethical Committee of Canton Ticino (CE 3030-2016-00193). The patients/participants provided their written informed consent to participate in this study. Written informed consent was obtained from the individual(s) for the publication of any potentially identifiable images or data included in this article.

## Author Contributions

All authors listed have made a substantial, direct and intellectual contribution to the work, and approved it for publication.

## Conflict of Interest

The authors declare that the research was conducted in the absence of any commercial or financial relationships that could be construed as a potential conflict of interest.

## APPENDIX 1: List of questions used in the interviews

1. How would you describe your overall experience of the program?
2. How would you describe your relationship with the residents who also took part to the program?
3. How would you describe your relationship with staff members?
4. Did this program have some effect on your feelings while listening to music?
5. Using a 1-10 scale, how would you evaluate your singing ability? Could you explain the reasons of that evaluation?
6. Using a 1-10 scale, how would you evaluate your ability to keep a steady rhythm? Could you explain the reasons of that evaluation?
